# Choosing a culture medium for SCNT and iSCNT reconstructed embryos: from domestic to wildlife species

**DOI:** 10.1186/s40781-017-0149-1

**Published:** 2017-11-10

**Authors:** A. Cordova, W. A. King, G. F. Mastromonaco

**Affiliations:** 1 Department of Biomedical Sciences, Ontario Veterinary College, University of Guelph, Guelph, Ontario Canada; 2 Reproductive Physiology, Toronto Zoo, Scarborough, Ontario Canada

**Keywords:** Somatic cell nuclear transfer, in vitro culture, Culture media, Nutrients, Embryos, Domestic species, Wildlife species

## Abstract

Over the past decades, in vitro culture media have been developed to successfully support IVF embryo growth in a variety of species. Advanced reproductive technologies, such as somatic cell nuclear transfer (SCNT), challenge us with a new type of embryo, with special nutritional requirements and altered physiology under in vitro conditions. Numerous studies have successfully reconstructed cloned embryos of domestic animals for biomedical research and livestock production. However, studies evaluating suitable culture conditions for SCNT embryos in wildlife species are scarce (for both intra- and interspecies SCNT). Most of the existing studies derive from previous IVF work done in conventional domestic species. Extrapolation to non-domestic species presents significant challenges since we lack information on reproductive processes and embryo development in most wildlife species. Given the challenges in adapting culture media and conditions from IVF to SCNT embryos, developmental competence of SCNT embryos remains low. This review summarizes research efforts to tailor culture media to SCNT embryos and explore the different outcomes in diverse species. It will also consider how these culture media protocols have been extrapolated to wildlife species, most particularly using SCNT as a cutting-edge technical resource to assist in the preservation of endangered species.

## Background

Somatic cell nuclear transfer (SCNT) has steadily developed over the past few decades, and its successes are evident in a variety of fields. One promising outcome is the production of transgenic animals for biomedical research and livestock production [1]. In the pig, for example, SCNT has been used to create piglets with specific genetic modifications applicable as biomedical models for humans [2, 3]. Meanwhile the goat has been a valuable species for the production of animals [4, 5] secreting transgenic proteins in milk [6]. In the area of conservation, reproductive technologies are being currently developed with the hope of preserving critically endangered animals (see review [7, 8]). Since SCNT technology preserves the entire genome from individuals, particularly non-reproductive or deceased animals, it can be a valuable tool for preserving genetic material from at-risk species. SCNT has been successfully performed in a variety of domesticated species including cattle [9], pigs [10], goats [11, 12], mice [13], cats [14], horses [15], rabbits [16], rats [17], dogs [18] and ferrets [19]. However, the successes have limits: blastocyst rates and subsequent pregnancy and birth rates remain low compared to other embryo production techniques. Moreover, these embryos are easily affected by culture conditions and arrest at various stages of development, including after birth, due to certain developmental defects [20].

It is well known that in vitro fertilized (IVF) embryos are less competent compared to embryos produced in vivo [21, 22]. In mammals, these embryos are subjected to altered, suboptimal environmental conditions when compared to their in vivo counterparts [23]. In spite of the efforts made to improve these suboptimal conditions, in vitro produced (IVP) embryos are still significantly affected, whether measured in terms of morphology, apoptotic index, blastocyst rate, cryotolerance, gene expression or postnatal growth [22]. Reconstructed SCNT embryos have been consistently found to be less competent than IVF embryos [24, 25] and efforts to optimize culture conditions may not have been sufficient. The processes underlying reprogramming in SCNT embryos and IVF embryos are different, and thus, media must be developed according to the special physiology and metabolic requirements of SCNT embryos.

This review focuses mainly on the improvements made in culture conditions with the aim of supporting the reconstructed SCNT embryo in domestic species, reviewing as well the extrapolation of these models to the special needs of endangered wildlife species.

### Somatic Cell Nuclear Transfer (SCNT)

In general terms, SCNT involves the removal of the nucleus and polar body from an oocyte (cytoplast) that then receives a nucleus from an external somatic cell (donor nucleus). When oocytes are scarce and a competent species-specific cytoplasm is unavailable, such as in the case of endangered species, interspecies SCNT (iSCNT) is a valuable approach. This variation uses the recipient cytoplast and donor nucleus derived from a related and abundant species (for review [26]). Although the most commonly used recipient cytoplasts have been bovine and rabbit for their demonstrated capacity to reprogram somatic cells as well as their availability [27–30], some studies have used pig [31], goat [32] and sheep [33], among others.

The standard SCNT technique includes three steps: 1) enucleation of the oocyte, 2) insertion of the nuclear donor cell, and 3) activation of the reconstructed embryo [1]. Each of these delicate steps influences the likelihood of producing a competent reconstructed embryo. Details of the SCNT technique have been well described by different authors and are worth reviewing with attention [34–36].

Studies are primarily concerned with developing robust, simple, and repeatable SCNT techniques. Indeed, researchers have investigated and improved each of the key steps: timing and method of enucleation [37, 38], fusion parameters [39], timing and method of oocyte activation [40–42], effects of DMSO on reprogramming the nucleus or inhibition of cytokinesis [43] and finally, culture conditions.

Successful reconstruction of the SCNT embryo depends on intrinsic (e.g. maturation of the cytoplast and reprogramming status of the donor nucleus) and extrinsic factors (e.g. culture conditions). In this review, we will describe the intrinsic conditions briefly below, and focus primarily on extrinsic culture conditions affecting both blastocyst and postnatal development.

### Intrinsic factors: Maturation of the recipient and reprogramming status of the donor

#### Maturation status of the recipient cytoplasm

Maturation status of the recipient oocyte is one of the key steps in SCNT. The recipient cytoplasm will control the donor nucleus’ epigenetic reprogramming, allowing the acquisition of an undifferentiated embryo-like chromatin status compatible with embryonic gene activation and development [44, 45]. In vivo matured cytoplasts have been shown to be considerably better in terms of blastocyst production, pregnancy rate and embryo survival [46]. Although, contradictory results have reported not observing any differences in capacity when comparing ovulated or in vitro matured oocytes in porcine SCNT [47].

Even if oocytes matured in vitro are able to offer some advantages over ones matured in vivo in terms of costs and better control over the maturation process, in vitro processes require complex maturation media and conditions to prepare the cytoplasm for the arriving differentiated cell nucleus, which can be more challenging. A variety of systems have been tested in diverse species and basic media have been used by different studies to prepare recipient cytoplasts: TCM 199 supplemented with fetal calf serum and hormones (cattle: [48]; mouse, sheep and goat: [49]), NCSU supplemented with hormones and follicular fluid (pig: [50]), and a mix of TCM199 media supplemented with hormones and follicular fluid (pig: [51]) as well. Moreover, considerable variations have been adapted to the above-mentioned conditions. Addition of leptin [52] or melatonin [53] to the maturation medium has been investigated. NCSU-23 medium supplemented with leptin increased significantly the percentage of oocytes reaching metaphase II and further augmented the blastocyst rate and cell number in SCNT embryos [52]. Supplementation with melatonin was demonstrated to decrease the levels of reactive oxygen species (ROS) in early SCNT embryos, but did not reduce the proportion of apoptotic cells in blastocysts nor increased blastocyst rate compared to controls [53]. In pigs, supplementation of in vitro maturation (IVM) culture with lanosterol improved preimplantation development of SCNT embryos, possibly by elevating lipid content of oocytes or regulating genes involved in the cholesterol biosynthetic pathway in cumulus cells or those involved in lipid metabolism and apoptosis, with a positive effect on blastocyst development [54]. Furthermore, the addition of rapamycin [55], colcemide [56], or coculture with denuded oocytes and their oocyte secreted factors [57, 58] were also tested during IVM in order to stimulate preimplantation development in SCNT embryos.

#### Nuclear donor reprogramming status

SCNT is meant to transform already differentiated somatic cells (donor cell) into totipotent blastomeres by reprogramming the external nucleus to give rise to an embryo. This reprogramming process is often stochastic and most probably responsible for the developmental defects of SCNT embryos [20]. Cell donors used to reconstruct embryos by SCNT vary: ear fibroblasts [24, 59], mammary gland cells [60], fetal fibroblasts [61, 62], cumulus cells [13], and oviduct/uterine epithelial cells [63] have been used from live to post-mortem donors [64]. No particular donor cell has been determined to be superior in overcoming the reprogramming issues faced in SCNT [20] across all species and conditions, but success can vary according to the species chosen [13, 65]. Suitable donor cells are most likely least differentiated and express epigenetic marks suitable for reprogramming. In order to improve the donor cell status and SCNT technique, a number of parameters have been tested, including cell type and passage number, cell cycle stage, and method of delivery to the enucleated oocyte [59, 66–71]. Incomplete or delayed reprogramming of the donor nucleus will result in abnormal embryo development including altered metabolic, energetic, and other physiological requirements.

A well-documented approach has been to treat the donor cells with pharmacological agents to remove some epigenetic marks before SCNT, improving the cell’s ability to be fully reprogrammed by the recipient cytoplast. The study by Enright et al. (2003) showed that treating bovine donor cells with Trichostatin A (TSA) increased the development of SCNT embryos to the blastocyst stage by increasing histone acethylation patterns [72]. However, treatment with TSA can have other outcomes: high doses of TSA can be detrimental for the embryo causing chromatin breaks and apoptosis [73]. Enright et al. (2003) used the lowest concentration of TSA to improve blastocyst formation [72]. In other studies, it has been shown that although lowering methylation levels by treatment with 5-aza-2'-deoxycytidine (5-aza-dC) does not always improve SCNT embryo development [72, 74, 75], it does decrease methylation status in the donor cell [72]. Results have varied among studies and species treated [76, 77], and lack of enhancement of SCNT embryo development is probably due to the differences in time of exposure and concentration of the drug during treatment. In any case, the question of how many epigenetic marks to remove remains unsolved: they confer stability to the embryo throughout its development, and any changes will be reflected in the expression profile of specific genes at later stages of development [20] affecting blastocyst quality and further postnatal development.

### Extrinsic factors: culture conditions for SCNT embryos

Low SCNT efficiency [43, 78] has been attributed to a variety of reasons: loss of donor cell nucleus’ ability to resume normal embryonic development as zygotic division progresses [48]; aberrant/incomplete reprogramming of the donor nucleus [79]; asynchrony between the donor nucleus and its recipient oocyte [80, 81], genetic make-up and age/senescence of the donor nucleus [82]; suboptimal oocyte activation [83]; and in vitro culture (IVC) conditions [84]. In IVF or SCNT embryos, the key steps of their developmental process occur under suboptimal in vitro conditions: first cleavages, timing of embryonic genome activation, compaction, and blastocyst formation may all be adversely affected by the culture media and conditions [85]. For wildlife species, whose reproductive processes and embryonic development have not been completely elucidated, tailoring culture conditions for SCNT embryos is still more challenging. Beyond the differences between IVF and SCNT, techniques developed for well-understood domestic species may need significant modification for non-domestic species. In the next pages, we will briefly describe the most commonly used IVC media used by different researchers for SCNT embryos in domestic animals and wildlife species including the most significant results in terms of blastocyst, apoptosis and pregnancy rate. Table 1 summarizes the different media used according to the species studied.

**Table 1:** Different base media used for SCNT and iSCNT embryo culture in domestic and wildlife species

Culture Medium Name	Acronym	Species
Tissue Culture Medium 199	TCM199	Cattle^a^, domestic goat^a^, macaque^b^, cat^a^, wild cat^b^
(m) Charles Rosenkrans	(m) CR1, (m) CR2	Cattle^a^, banteng^b^, yak^b^, domestic goat^a^, argali sheep^b^
(m) Synthetic Oviductal Fluid	(m) SOF	Cattle^a^, bison^b^, buffalo ^b^, gaur^b^, domestic goat^a^, sheep^a^, sheep^b^, ibex^b^, argali sheep^b^, rhesus monkey^b^, crab-eating monkey^b^, cynomolgus monkey ^b^, dog^a^, gray wolve ^b^, marbled cat^b^, cat^b^, cheetah^b^, cat^a^
North Carolina State University	NCSU	Pig^a^
Bovine Embryo Culture Medium	BECM	Pig^a^
Porcine Zygote Medium	PZM	Pig^a^, domestic goat^a^, dog^a^
G-series culture media	G1.5/G2.5, G1/G2	Cattle^a^, rhesus monkey^a^, dog^a^
M16 medium	M16	Mouse^a^
Potassium Supplemented SOM (Simplex Optimised Medium)	KSOM	Domestic goat^a^, mouse^a^
Chatot, Ziomec, Bavister Medium	CZB	Mouse^a^
B2 Medium	B2	Rabbit^a^
Earle’s Balanced Salt Solution	EBSS	Rabbit^a^
Connaught Medical Research Laboratories 1066	CMRL 1066	Rabbit^a^, cynomolgus monkey^b^
Hamster Embryo Culture Medium	HECM	Rhesus monkey^a^, cynomolgus monkey^b^
Tyrode’s Medium	-----	Cattle^a^

^a^SCNT, ^b^iSCNT. m: modified

### Cattle

Bovine embryo culture systems have been most extensively studied of all the livestock species; from simple to chemically defined compositions. Over the years, studies have utilized diverse base media, such as TCM199 [86], modified Tyrode’s medium [87], CR medium [88], SOF medium [89] and so on, to improve developmental rates in IVF embryos. Researchers have also compared conventional media with the aim of best supporting bovine SCNT in vitro development.

The study performed by Choi et al. (2002), for example, compared modified CR2aa (mCR2aa) and modified SOF (mSOF) with the addition or sequential addition of macromolecules (BSA, PVA or FBS) [90]. They demonstrated that mSOF medium provided better conditions for 2-cell embryo, morula and blastocyst development, and the addition of macromolecules (BSA followed by FBS) in mSOF was the best combination to obtain higher blastocyst rates, total cell number and inner cell mass (ICM) cell number. Unfortunately, results were not reflected in pregnancy outcomes [90]. In addition, the variable composition of BSA or FBS makes it difficult to specifically determine their potential benefits on preimplantation development of SCNT embryos [84, 91]. Serum/BSA-free culture media for SCNT embryos are important to explore to avoid postnatal complications associated with exposure to these proteins in vitro [92, 93]. Medium G1.2/G2.5 was originally developed to respond to the changing requirements of the cultured IVF embryo, taking into account the change of energy requirements throughout their first embryonic stages and after genome activation [94]. Xiong et al. (2014) compared mSOF and G1.5/G2.5 media, finding that SCNT embryos have a preference for G1.5/G2.5 [24] as evidenced by low apoptosis rates in blastocysts (decreased apoptotic blastomeres, apoptotic index and relative expression of *HSP70* and *BAX*). In contrast, IVF embryos seemed to prefer mSOF when evaluating the same parameters [24]. This study confirmed the research done by Wang et al. (2011) that demonstrated the efficacy of G1.2/G2.2 sequential media for supporting bovine SCNT embryo development and subsequent calving rate when compared with mSOF alone [95].

Success with bovine IVP has led to the bovine cytoplasm being widely used to support reprogramming of somatic cells from different species and give rise to embryos, especially for endangered species. iSCNT with bovine cytoplasm has been used to reconstruct non-domestic bovine embryos, such as gaur [96], bison [27], buffalo [97–99], and banteng [100] (for more extensive review see Mastromonaco et al. [7]). The study performed by Sansinena et al. (2005) reconstructed embryos using bovine cytoplasm and male or female banteng adult fibroblasts and cultured them in CR1aa medium with 5% serum added until day 7 of development when the embryos were transferred into cow recipients. Results showed blastocyst rates ranging from 15 to 28% and a 17% pregnancy rate detected on day 30 of gestation when using male bantengs adult fibroblasts as donors [100]. At days 60 and 90 of pregnancy, detection of the fetus was lost. Seaby et al. (2013) by other hand, investigated the development of iSCNT embryos using bison adult fibroblasts and bovine cytoplasm in mSOFaa with 2% serum based on their bovine IVF system. Although blastocyst rates were 19 to 33%, these bison iSCNT embryos had a greater number of apoptotic cells when compared to their IVF and cattle SCNT counterparts [27]. Several studies also can be mentioned on the addition of antioxidants and the use of coculture for its antioxidant capacity to the culture media for SCNT and iSCNT embryos in domestic and wildlife species. Studies in buffalo have used coculture with BRL cells for 48 hours and then buffalo oviduct cells, obtaining blastocyst rates ranging from 26 to 60% using different cell types donor (either fetal fibroblasts, cumulus cells or oviduct cells) [99]. The study suggests that the increased blastocyst developmental rates observed may also be due to the fact that fetal fibroblasts were used as cell donors over somatic adult cells. Lu et al. (2005) used granulosa cells as coculture for their buffalo and bovine SCNT and iSCNT embryos, but blastocysts rates were low; only 4.5% for bovine-buffalo iSCNT blastocysts and 13.3% for buffalo-bovine. Meanwhile, only 3% blastocyst rate was observed in buffalo and 11.9% in bovine SCNT groups. The low blastocyst rate observed in buffalo SCNT highlights another important factor involved: oocytes from different species have different developmental competencies as exhibited by the results using buffalo versus bovine oocytes in this study [97]. The study performed with yak-bovine iSCNT embryos used a sequential medium consisting of CR1aa supplemented with BSA along with cumulus cell coculture, yielding 30 to 34% blastocyst rates [101]. Despite the abnormal composition of ICM and trophoectoderm (TE) cells and abnormal expression of embryo-specific genes (both potentially responsible for the post-implantation problems observed), some pregnancies and births resulted [101]. In 2010, the study performed by Srirattana et al. used mSOFaa followed by coculture with bovine oviduct epithelial cells in mSOFaa for 8-cell stage cattle or buffalo SCNT and parthenogenic embryos. Blastocyst rates obtained were not significantly different among the different SCNT embryos, ranging from 40 to 46% in cattle and 26 to 30% in buffalo despite the different donor cell type used (either fetal or ear fibroblasts, granulosa or cumulus cells) [102]. Two years later, the same group evaluated the effect of the addition of TSA on cloning efficiency, using mSOFaa medium for 2 days followed by a coculture with oviduct epithelial cells (in mSOFaa medium) for 5 days in gaur-bovine iSCNT [96]. The results showed higher blastocyst rates (33 to 37%), and although multiple early pregnancies were detected, only one calf was born. Coculture with somatic cells were introduced to counteract the ROS produced in the culture medium. Other factors with antioxidant properties were also evaluated to improve bovine SCNT embryos, such as vitamin C [103] and 3-hydroxyflavone [104]. The addition of vitamin C positively affected ROS formation, as well as cleavage, morula formation, and index of apoptosis in blastomeres [103] although these results were not translated into higher blastocyst and hatching rates. In contrast, 3-hydroxiflavone showed improved results with a positive effect on both cleavage rate 24 hours post activation and blastocyst rate [104], as well as decreasing levels of intracellular ROS and pro-apoptotic candidate genes [104].

### Pig

Developmental competence of porcine oocytes/embryos derived in vitro has been significantly lower than those produced in vivo, reflecting the suboptimal culture conditions for porcine IVF embryos [41]. Studies in pigs provide good examples of the detrimental effects of culture conditions on in vitro embryo development. Therefore, introducing porcine IVF culture conditions to porcine SCNT embryos immediately presents a challenge for this technique. There are many issues with porcine IVP systems (either IVM or IVC), and despite the efforts, IVC conditions have not been completely optimized in this species [105–107]. For this reason, and in contrast to SCNT embryos from other species, porcine SCNT embryos have been usually transferred to the oviduct as early as the one cell stage. Still, developmental rates of pig SCNT embryos using in vivo or in vitro matured oocytes have been low [70, 108]. To improve pig IVP, several culture media have been developed for this species, including NCSU-23 and NCSU-37 media [109, 110], BECM-3 medium [109, 111], and PZM-3, PZM-4 and PZM-5 media [109, 112]. These were also used as basic media for SCNT embryos, and probably contributed to their low developmental potential in the same manner as for IVF embryos. Studies supplementing mNCSU medium with FBS (10%) on Day 4 showed an increase in SCNT blastocyst rate formation as well as total and ICM cell numbers [78]. However, the expression of the pro-apoptotic gene *BAX* was found elevated in SCNT blastocysts compared to their in vivo counterparts after supplementation with 10% FBS [78]. These results demonstrate once again the biphasic effect of FBS; on one side increasing embryonic development rates and on the other side altering gene expression, as shown previously when studying IVF embryos [111, 113, 114].

In pigs, glycoproteins have also been supplemented in the IVC media. Lee et al. (2013) evaluated the addition of granulocyte macrophage colony-stimulating factor (GM-CSF) in PZM3 medium on SCNT embryos demonstrating that the addition of this glycoprotein can fully support embryo development to term in pigs [115]. Moreover, an increase in blastocyst stage embryos as well as total cell number was observed [115]. Similarly, epidermal growth factor (EGF) supplementation has been demonstrated to increase post-activation cleavage rate as well as cell number of porcine SCNT preimplantation embryos [116]. In this study, EGF and its receptor mRNA were found to be expressed in pig SCNT embryos for the first time [116].

Porcine SCNT preimplantation embryos might be affected not only by culture medium but also by oxygen concentrations, which augment ROS and affect blastocyst formation. A well-defined medium can compensate for high oxygen concentrations and deleterious ROS [109]. Moreover, studies have shown that porcine SCNT embryos have higher apoptotic incidence compared to IVF embryos [117]. The study performed by Yamanaka et al*.* (2009) was able to demonstrate that modified PZM5 decreased apoptosis incidence in SCNT embryos, positively impacting blastocyst formation rates [118]. Additionally, as coculturing with feeder cells can also decrease ROS from culture media, Ju et al. (2012) cultured reconstructed porcine embryos with a monolayer of mytomicin C-inactivated cumulus cells (iCC) [51]. Interestingly, with this system, SCNT blastocyst rate increased significantly with a corresponding decrease in apoptotic incidence [51]. Vitamin C was also tested. The study performed by Huang Y et al. in 2011 demonstrated positive results after the addition of Vitamin C to PZM3 medium for as little as 15 hours post-activation with an improvement in blastocyst development and pregnancy rates [119]. The addition of different concentrations of Vitamin C in this study was able to improve blastocyst SCNT rates (from 29 to 36% compared to 11.5% in non- supplemented group) and increase *OCT4* and *SOX2* gene expression levels, which would demonstrate the capacity of this antioxidant to enhance blastocyst quality by increasing these pluripotency-related genes. Finally, when blastocysts were transferred, the Vitamin C supplemented group had 45% of pregnancies detected compared to 33.3% in the control group. Out of these, full term development was also higher in the group supplemented with Vitamin C (20% versus 6.7% control) [119].

Studies have used pig oocytes as recipients to reconstruct dog [120, 121], cattle [120, 122], mouse [120, 122], rat [31], chicken [122], and whale iSCNT embryos [123], among others. In fact, it has been postulated that the pig oocyte could be used as a universal recipient due to its high demethylation capacity that is able to support the initiation of nucleoli formation and subsequent early embryo development in several species [120]. Different media were used despite the fact that pig oocytes were the recipients in all cases. In 2011, for example, Lagutina et al. used mSOFaa when using pig oocytes as recipients for multiple species. In the study by Gupta et al. (2013), NCSU medium designed for pig oocytes was able to support embryonic development of different donors such as bovine, rodents and chicken [122]. Studies to reconstruct pig iSNT embryos using pig as nuclear donor are scarce. In 1999, Dominko et al. used CR1aa medium to support porcine-bovine iSCNT embryos with 14.3% blastocyst rate [124]. In 2007 Uhm et al. produced pig iSCNT embryos with cattle oocytes using CR1aa supplemented with BSA for IVC with a very low blastocyst rate (5.5%) [125]. More recently, Lagutina et al. (2011) used mSOFaa medium for pig iSCNT embryos with cattle oocyte recipient. When using rabbit oocyte as recipients, the medium used was Menezo B2 10% FBS [120]. Neither of these two cytoplasms nor the IVC media used showed the ability to support the pig nucleus through embryonic genome activation.

### Goat and Sheep

In goats, abnormalities associated with SCNT technique have not been widely reported, probably because goat embryos spend minimal time under IVC conditions [5]. As with pigs, transfer of goat SCNT embryos are performed as early as the 1-2 cell stage in order to avoid IVC as much as possible (maximum of 2 days) [11]. This is a good practice for improving development rates, but transferring embryos into the oviduct requires surgery, while allowing embryos to develop into later stages for intrauterine transfer is more convenient and less invasive. Still, little research has been carried out in goat IVP systems since blastocysts cultured in vitro have difficulties developing to term [49]. For these SCNT embryos, different basic culture media have been used including SOF [126], CR1 [127, 128], and TCM199 [129]. Furthermore, the chemically defined medium G1/G2 has been most often used to produce viable offspring [5, 11]. Tang et al. (2011) compared G1/G2 to mSOF-BSA medium, or mSOF-BSA supplemented with FBS. These researchers determined that the inclusion of FBS in the mSOF medium improved the rates of hatching of goat SCNT blastocysts, but no differences were found in terms of 2-cell, 8-cell, morula or blastocyst rates [49]. On the other hand, the use of sequential two-step media (G1.2/G2.2 and SOF1/SOF2;SOF1 with FBS and glucose) showed that G1.2/G2.2 improved SCNT blastocyst rates, but SOF1/SOF2 improved pregnancy and live birth rates [130]. The two-step culture system studied by Kwong et al. (2012) consisted of KSOMaa with increased glucose concentrations from day 2 of culture. As in other species, this study found that augmenting glucose at later stages of embryo development promoted a significant increase in goat SCNT blastocysts and hatched blastocysts, compared with a steady-state low glucose concentration throughout culture [39].

Ground breaking research in sheep cloning date as far back as the late 1980’s with the study from Willadsen (1986) [131] and others [46, 132–134]. Before Dolly’s birth, nuclear transfer of adult cells was generally considered as a procedure where rigid mechanisms had to be reversed. After her birth this vision changed and studies of clones from adult cells of a variety of species were developed rapidly (for review see Wilmut et al. [135]). Already, Loi et al. (1997) worked in sheep embryo cloning using oocytes and blastomeres as karyoplasts [133] suggesting that the in vitro culture medium used (CZB) could have been less effective than the mSOF used by McLaughing et al*.* in previous studies [132]. Later on, Hua et al. worked with domestic sheep using mSOF medium supplemented with essential/non-essential amino acids, glutathione and EDTA for their ovine-bovine iSCNT embryos [30]. Despite the high proportion of low embryo quality found, this study was able to produce 24.6% blastocysts, which the authors consider may be mostly reflection of the donor cell used for iSCNT (sheep fetal ear skin tissue) [30]. Peng et al. (2008), on the other hand, supplemented mSOF culture medium with alpha tocopherol aiming to decrease apoptosis and possibly improve SCNT embryo production. Their results showed no differences in blastocyst rate or cell number, although apoptotic index was decreased in the treated embryos [136]. Wen et al. (2014) also used mSOF to culture embryos after treating them with deacetylase inhibitors that resulted in blastocyst formation ranging from 10 to 24% [137]. Similar data to this study was found previously by Choi et al. (2013) in which the same medium was used resulting in blastocyst rates of 19 to 31% [138].

As for wild goat species, iSCNT has been successfully carried out in the endangered ibex using domestic goat cytoplasts [139]. This study performed by Wang et al. (2007) supplemented mSOF with essential and non-essential amino acids, glutamine, BSA and serum for the ibex IVC medium and achieved a blastocyst rate of 11% (although less than goat SCNT and parthenogenic embryos) [139]. Moreover, in wild sheep, iSCNT has been used to reconstruct argali-sheep iSCNT embryos using both domestic ovine and bovine cytoplasm [33]. Sheep methodology closely followed protocols used for the successful cloning of Dolly [60]. Both the argali-sheep and argali-bovine iSCNT embryos were cultured in CR1aa medium, varying only the times of culture in vitro [33]. From all reconstructed argali-bovine iSCNT couples, 77% successfully fused, and from those 78% developed to 8-16 cells stage embryos but only 2 (1.56%) survived to the blastocyst stage. Meanwhile, when reconstructing argali-sheep iSCNT embryos from 15 oocytes, 12 were fused and 10 developed to 16-32 cells, but development then arrested [33]. More recently in 2014, the study by Pan et al. (2014) used mSOF supplemented with BSA to culture argali-sheep iSCNT embryos. After 2 days of culture, 5% FBS was added until day 8 [140]. From all embryos fused, only 15.7% developed to blastocyst, a significantly lower percentage than their SCNT counterparts (sheep-sheep SCNT, 20.4% and 22.1%).

### Mouse and Rabbit

Mouse SCNT embryos are very sensitive to IVC conditions [141] as early as the one-cell stage and have different requirements throughout their culture in vitro [80]. CZB medium (supplemented or not) was reported to successfully support early development of mouse SCNT embryos [13, 40]. Additionally, Chung et al. (2002) discovered that mouse SCNT embryos needed glucose supplementation during IVC, unlike their parthenogenic or IVF counterparts [80]. This glucose preference demonstrates their altered metabolism in vitro. In fact, the beneficial effect of glucose on SCNT early embryos suggests a different glucose metabolism and method of use as an energy substrate throughout the culture period [80]. In this respect, Dai et al. (2009) developed a sequential “D medium” consisting of M16 and KSOM. It is known that culture condition requirements change according to the embryo’s stage of development. In the mouse, this change occurs at the 2-cell stage, when maternal zygotic transition occurs [142]. KSOM is a well-studied medium for IVF embryos, but has not been shown to support mouse SCNT embryos before the 2-cell stage [80]. The sequential medium used in Dai et al.’s study, introducing KSOM after the first cleavage, yielded the highest blastocyst rates reported, with normal rates of apoptosis per embryo [143]. In mouse SCNT embryos, the addition of antioxidants such as melatonin was also studied. In fact, Salehi et al. (2014) supplemented SCNT embryo culture with different concentrations of melatonin in KSOM finding that its addition was able to improve blastocyst rate [144]. Melatonin was added as free radical scavenger [145] and for its capacity to stimulate antioxidant enzymes in the culture medium [146]. With the same aim, the addition of Vitamin C in KSOM medium was tested by Mallol et al. later on, resulting in the improvement of blastocyst developmental rates up to 45.9% when treated for 120 hours compared to non-treated reconstructed embryos (26.7%) [42].

Successful rabbit clones were first reported by Chesne et al. (2002) where reconstructed embryos were cultured in B2 medium supplemented with FBS [16]. Since then, only a few successful live births have been reported, but most of them died within the first 3 weeks after birth [147–150]. Among these studies, most of the reconstructed embryos were transferred into a recipient oviduct for development and others were cultured in either B2 medium (supplemented with 2.5% FBS) or in EBSS medium. Moreover, Tian J et al. (2012) used EBSS complete medium for SCNT embryo culture and obtained good blastocyst rates ranging from 39 to 79% [151]; although these variations in outcome appeared to be mainly due to the donor nuclei used during SCNT. In rabbits, SCNT embryos were recently reconstructed after in vitro growth (IVG) and IVM of follicles, and subsequently cultured in supplemented CMRL-1066 medium [152]. Although oocytes grew and matured in vitro, their cleavage and blastocyst rates after fusion and activation were similar to those matured in vivo [152]*.* This innovative result suggests that IVG-IVM oocytes have developmental competency and the resulting SCNT embryos were reprogrammed and supported by the medium in which they were cultured.

### Macaque

Few SCNT studies exist in non-human primates (NHP). iSCNT, on the other hand, has been considered a good alternative: NHP research can be costly, and using cytoplasm from other well-studied species can reduce costs. For either SCNT or iSCNT, different media have been tested; HECM (Rhesus macaque SCNT: [153]); SOF (Crab-eating macaque iSCNT: [154]); CMRL-1066 and M199 (Cynomolgus macaque iSCNT: [29]). In 2006, Zhou et al. used HECM as primate SCNT embryo culture medium in their work to compare two SCNT methods in rhesus monkeys [153]. Their results were encouraging, ranging from 12 to 24% blastocyst rates depending on the SCNT method used but with the same culture medium [153]. In 2008, the group of Lorthongpanich et al. studied the feasibility of reconstructing embryos using cynomolgus monkey – bovine iSCNT and varying culture medium and timing [154]. They based their experimental embryo culture design on two important factors: 1) the cytoplasmic mRNA of an embryo up to the eight-cell stage is driven by the recipient cytoplasm instead of the donor nucleus [154], and 2) embryos synthesize their own mRNA material from the eight-cell stage onwards (MZT in certain species) [155]. Even if HECM and mSOF media were tested at different time periods (before and after MZT), the results did not show any enhancement in embryo growth [154]. This may have been due to reasons not related directly to the culture medium but the genetic distance between primate donor and bovine recipient. Kwon et al. (2011) used mSOF culture medium for 10 days under different oxygen concentrations to produce rhesus monkey-bovine iSCNT embryos, but the resulting blastocyst rates were extremely low. In macaques in general, the most suitable culture condition for these iSCNT embryos is not yet clear, but this study suggests that the medium is crucial, while oxygen and temperature during culture may not be as important in this species [28]. The research performed by Yamoshi et al. (2013) tested rabbit cytoplasts as recipients for cynomolgus monkey nuclei [29] and used culture media previously studied for rabbit embryo production in vitro, but optimized for this macaque [156]. This research group found that the blastocyst rate was clearly improved when using mCMRL-1066 (17%) compared to M199 media (0%, all embryos arrested at 4 cells). Modified CMRL-1066 is a more complex culture medium than M199, containing lactic acid, pyruvic acid, FBS and coenzymes. More importantly, this study suggests that iSCNT embryos prefer the embryo culture medium supportive of the nuclear donor for preimplantation development [29]. Some other studies have tested different types and timing of sequential media, which should be taken into account since it impacts the final result. For example, the study by Ng et al. (2004) used small periods of culture in IVF-20, G1.1 and G2.2, respectively. Although the embryos were transferred three days after SCNT, none of them proceeded past 60 days of gestation. Meanwhile, Simerly et al. (2004) used longer periods with the same media (G1 and G2) and included also mSOF supplemented with fructose from the morula stage onwards [157]. Although the purpose of this study was not to evaluate embryo culture but the effect of different donor cell sources, it is expected that different culture media could have had an impact on early embryo development and subsequent post-implantation survival. In this case, embryo development seemed accelerated, but ultimately, low blastocyst rates were achieved and no evidence of sustained pregnancy was detected.

### Dog and Cat

The dog model can be particularly challenging due to their unique physiology, especially the difficulty of obtaining mature oocytes for successful SCNT, and lack of a good IVP system. The majority of canine SCNT studies have used oocytes matured in vivo; still, the production of good-quality blastocysts is low and this is partly due to suboptimal culture conditions. To minimize the effect of in vitro conditions, canine reconstructed embryos have been transferred into the oviduct immediately after SCNT [158, 159]. However, a potential solution was the use of in vitro matured non-canine oocytes (such as cattle) as recipient cytoplasm and canine somatic cells as donors [160]. iSCNT in canine has been somewhat successful, with the production of 8-16 cell stage embryos [160], although further investigation is necessary to increase embryo production and obtain viable offspring. Recently, Kim et al. (2015) used in vivo matured canine oocytes for SCNT, and tested three commonly-used culture media (mSOF, PZM-3 and G1/G2) for IVC. Their results showed improved cleavage (87.8%) and blastocyst rates (7.8%) when culturing with G1/G2 media, compared with mSOF (59.1% and 0% blastocysts) or PZM-3 (85% and 0% blastocysts) [161]. As for wildlife species, successful cloning has been performed in coyote where fused embryos were kept in TCM199 until transfer into female domestic dogs [162]. Endangered gray wolves were cloned successfully as well by iSCNT using domestic dogs as oocyte donors, but the zygotes produced were either transferred into recipients 4 hours after activation [64] or kept in mSOF until embryo transfer [163]. Pregnancies and live births resulted in both cases.

Felid SCNT has had some of the more successful outcomes. In this case, iSCNT is especially valuable for feline assisted reproduction given the rarity of wild oocytes [14, 164, 165]. Although cats have been considered difficult to clone, the study performed by Yin et al. (2005) succeeded using TCM199 supplemented with BSA as the embryo culture medium, with transfer to a recipient oviduct both at the one-cell stage after fusion, and at the two to four-cell stages [164]. Development was similar in rates recorded for African wild cat iSCNT and domestic cat SCNT embryos [165, 166] but embryo quality was reduced, suggesting that TCM199 with BSA is not the optimal medium to use for cat iSCNT embryos. Thongphakdee et al. (2006) used SOF with 10% serum to culture marbled cat iSCNT embryos and evaluated different oocyte culture times, fusion rates and reprogramming status [167]. This study did not report blastocyst development. However, Wen et al. (2003) did show higher blastocyst rate in cat-rabbit iSCNT embryos cultured in SOF with 10% serum (14.5%) compared to M199 with 10% serum (6.9%), even if blastocyst rates in cat-cat SCNT using the same media were not significantly different [168]. Other iSCNT embryos were developed to 2-4 cells and then transferred into the oviducts of recipient cats but no pregnancies were detected in either case. In addition, Moro et al. (2015) also used SOF with 2.5% serum in microwells to culture cheetah-cat isCNT or cat-cat SCNT embryos [169] testing individual or group culture. Results were very encouraging with increased cleavage (cat-cat: 87.6 to 98.2%; cheetah-cat: 87.2 to 96.7%) and blastocyst (cat-cat: 27.4 to 47.7%; cheetah-cat: 16.7 to 28.6%) rates when culturing in groups compared to individually. Despite the high cleavage rates, this study demonstrated that culturing in SOF with serum supported better blastocyst development when embryos were aggregated during culture.

SCNT is still undergoing significant research for feline species. The cat oocyte cytoplasm is fragile and easy to break during enucleation [168] making its manipulation more problematic. Therefore, in addition to the inherent problems with SCNT, in most of the cases, the culture conditions are not efficiently supporting the development of the embryo through to the blastocyst stage. In fact, the successful cloning reported in cat species arriving to term [170, 171] refers mostly to embryos transferred early to the oviduct, thereby avoiding prolonged exposure to in vitro culture media.

## Conclusions

Despite all the efforts to mimic in vivo conditions in vitro*,* and the improvements made in IVF culture media over the years, embryo development rates are still low in numerous species. Moreover, long-term developmental imprinted consequences have been observed in sheep and cattle [172, 173], as well as mouse [174], due to sub-optimal culture conditions.

The same challenges faced decades ago with IVF embryos and the creation of culture media able to support their development are now being faced with SCNT embryos. However, we can now see some promising areas to investigate.

First of all, the vast amount of information available pertains to IVF embryo culture, which is still being used as the closest reference despite the fact that SCNT embryo physiology and metabolism are altered and their environmental requirements in vitro are different from those required by IVF embryos. More research is still necessary to efficiently supplement SCNT media with factors, including antioxidants, to mitigate the damage, such as increased apoptotic indices and altered metabolic profiles, observed in SCNT embryos [117]. Moreover, the best option for some species, such as the pig, goat, dog and cat, has been to transfer the reconstructed embryo into the oviduct as early as possible avoiding in vitro conditions. Despite the great efforts over the years to create synthetic media that mimic the oviduct content, further studies in SCNT/iSCNT embryos are still required to elucidate the factor(s) that make the oviduct the suitable place for embryos to develop.

In better-studied species, sequential media seem to be providing improved results as they cater to the developmental needs of the embryo. In cattle, for example sequential media increase blastocyst and pregnancy rates [95]. Even the introduction of coculture has proven beneficial for its capacity to decrease ROS in media, although it is not in common use due to sanitary concerns and the known risks of large offspring syndrome (LOS) [175]. For goats and sheep, the best solution for improving blastocyst and pregnancy rates has been two-step culture media, with the addition of glucose for reconstructed embryos. For mice and rabbits, sequential media using M16 and KSOM improve blastocyst rates and lower apoptotic indices. However, continued research is necessary to fully account for SCNT embryo physiology in vitro.

Despite the advances in developing suitable culture media, successful in vitro preimplantation development does not necessarily reflect success after transfer [176]. In fact, SCNT embryos still have the highest rates of early pregnancy loss, late abortion, aberrant organogenesis and perinatal morbidity in all species studied [177, 178]. Successful postnatal survival even with established culture conditions is still low and needs to be improved. These problems are commonly associated with the epigenetic impact that culture conditions have on nuclear reprogramming and the given culture’s incapacity to support the reconstructed embryo.

To add to these issues, iSCNT overall is still problematic. Even if certain recipient cytoplasts are able to support the first mitotic divisions of the reconstructed embryo, the question remains whether these iSCNT embryos would be better supported in a culture environment specific for the recipient cytoplasm, or the foreign nucleus. Furthermore, it is still uncertain whether the preference for an optimal embryo culture medium will depend on the nuclear-donor or cytoplasm-recipient preference [29, 179, 180]. Thus, the genetic distance between the recipient and the donor must be taken into account when choosing the appropriate culture medium. In macaques, for example, iSCNT has not been as successful given the genetic distance between the receiving cytoplasm and the donor nucleus. Events, such as heteroplasmy or mitochondrial incompatibilities within iSCNT embryos, significantly impact nuclear-cytoplasmic events occurring at the time of embryonic genome activation (EGA) [181]; events that the culture medium should be able to support. Further investigations are still necessary to understand how IVC media would influence iSCNT and their capacity to surpass EGA [120].

Extrapolation from IVF embryo culture media to SCNT embryos is complex due to the inherent differences in embryo requirements and physiology, especially in wildlife species for which so much is still unknown. Finding the right culture medium conditions includes not only supporting higher numbers of blastocysts, but also supporting post-implantation survival and viable offspring. Despite all the efforts made, the existing data on IVC media and conditions used to support SCNT and iSCNT embryos are still contradictory, but crucial. Different studies are still necessary to continue optimizing and supplementing culture media able to support these reconstructed embryos through their full development in domestic animals with the potential to apply them later on in wildlife species.
